# Housing and road transport modify the brain neurotransmitter systems of pigs: Do pigs raised in different conditions cope differently with unknown environments?

**DOI:** 10.1371/journal.pone.0210406

**Published:** 2019-01-16

**Authors:** Laura Arroyo, Daniel Valent, Ricard Carreras, Raquel Peña, Josefa Sabrià, Antonio Velarde, Anna Bassols

**Affiliations:** 1 Departament de Bioquímica i Biologia Molecular, Facultat de Veterinària, Universitat Autònoma de Barcelona, Cerdanyola del Vallès, Spain; 2 IRTA, Animal Welfare Subprogram, Veïnat de Sies, s/n, Monells, Spain; 3 Servei de Bioquímica Clínica Veterinària, Facultat de Veterinària, Universitat Autònoma de Barcelona, Cerdanyola del Vallès, Spain; 4 Departament de Bioquímica i Biologia Molecular, Facultat de Medicina, Institut de Neurociències, Universitat Autònoma de Barcelona, Cerdanyola del Vallès, Spain; Queens College, UNITED STATES

## Abstract

How housing and transport conditions may affect welfare in porcine production is a leading topic in livestock research. This study investigated whether pigs present a different neurological response to management conditions and to ascertain whether pigs living partially outdoors cope differently with road transport-associated stress. Twenty-four female pigs were divided in two groups: one living indoors (ID, n = 12) and the other housed combining indoor conditions with 4 hours per day of outdoor pasture (OD, n = 12). After one month, one set of animals from each housing condition were driven in a truck to the slaughterhouse in low-stress conditions (5 min drive, no mixing groups, soft management, LS group, n = 12) or high-stress conditions (2 hours drive, mixing groups, harsh management, HS group, n = 12). At the slaughterhouse, blood was collected, and the prefrontal cortex (PFC) and the hippocampus (HC) dissected. OD pigs had lower serum haptoglobin and increased dopaminergic pathway (DA-system) in the PFC, suggesting that living outdoors increases their wellbeing. HS conditions increased serum creatine kinase (CK) and affected several brain pathways: activation of the noradrenergic (NA-system) and DA -system in the PFC and the activation of the DA-system and an increase in c-Fos as well as a decrease in brain-derived neurotrophic factor (BDNF) in the HC. The serotonergic system (5-HT-system) was mildly altered in both areas. There was an interaction between housing and transport in serum NA and the DA-system in the HC, indicating that living conditions affected the response to stress. Multivariate analysis was able to discriminate the four animal groups. In conclusion, this work indicates that housing conditions and road transport markedly modifies the neurophysiology of pigs, and suggests that animals raised partially outdoors respond differently to transport-associated stress than animals raised indoors, indicating that they cope differently with unknown environments.

## Introduction

How housing and transport conditions may affect welfare in porcine production is a leading topic in livestock research. Outdoor pig production is an alternative to conventional systems and it may improve the welfare of pigs since it reduces the chronic stress usually associated with intensive production [1]. The main welfare advantage of outdoor production are the lower degree of confinement, the increased cardiovascular fitness [2] and the lack of behavioural restriction due to the larger space, environmental enrichment (EE) and diversity [3,4]. Furthermore, outdoor rearing presents a better image to the consumer due to perceived better welfare and meat quality [5,6]. However, these animals might have to resist the rigours of the climate, like heat and cold [7]. An important issue is whether pigs reared in different housing systems cope differently (and show different stress response) in front of the same preslaughter factors such as lorry loading and unloading, transport, lairage at the slaughterhouse and driving to the stunning area [8]. Some studies suggest that pigs from EE systems can cope better and react less adversely to preslaughter factors than pigs from intensive housing systems [5,7,9]. Other studies showed no effect of EE on physiological stress indicators although the housing conditions influenced animal activity during transport [8,10], and a number of studies have also suggested that outdoor pigs experienced greater pre-slaughter stress [7,11,12].

In the central nervous system (CNS), noradrenergic, dopaminergic and serotonergic pathways are the most important and well characterized neurotransmitter (NT) systems underlying the response to stress, fear and reward [13–15]. Prefrontal Cortex (PFC) and hippocampus (HC) are recognized to play a role in the regulation of the stress response [16–20]. These areas have an indirect output to the hypothalamus, which acts modulating the final stress response through the sympathetic nervous system and the activation of the hypothalamic-pituitary-adrenal (HPA) axis, that release catecholamines and cortisol to the plasma, respectively [20,21]. NT systems, especially catecholamines (noradrenaline (NA); adrenaline (A); dopamine (DA) and their metabolites, homovanillic acid (HVA) and 3,4-dihydroxyphenyl acetic acid (DOPAC)), and the indoleamines (serotonin (5-HT) and its metabolite 5-hydroxyindoleacetic acid (5-HIAA)), play a significant role in the activity and interactions among those areas [13,22]. One of the downstream targets of these signalling pathways is the early-response gene c-*FOS* [23–26]. Sustained expression of c*-FOS* has been observed in a variety of stressful conditions and c-Fos is widely used as an estimate of neuronal activation [23,27], whereas activation of the ERK pathway has been proposed to signal the response to stress [28,29]. Not only stress but also positive conditions such as EE provoke changes in neurotransmitters and neurotrophic factors that are correlated to behavioural changes, learning and memory [30,31]. In laboratory animals, modifications in the monoamine NT profile linked to EE have been described. For example, EE alters the metabolism of DA and 5-HT in the PFC [32–35] and the serotonergic pathway in the HC [36]. The neurotrophin BDNF increases in EE [37,38], whereas usually decreases in acute and chronic stress [39] and depressive-like behaviour [40]. On the other hand, some studies state that EE attenuates pro-oxidative processes and triggers anti-oxidative defence mechanisms [38,41].

In pigs, studies have been addressed on brain adrenergic, dopaminergic and serotonergic NT profiles, related to stress-susceptible breeds [42], immobilization stress [43,44], prenatal stress [45], dominance and aggressiveness [46,47], tail biting [48] and fear [49]. However, no studies have been performed in pigs related to EE conditions.

Our research group has recently defined the changes provoked by management at the slaughterhouse in the pig dopaminergic and serotonergic neurotransmitter profile in several brain regions [49]. In the present study, we have analyzed the changes in monoamine NT profile after road transport-associated stress in pigs which have been living totally indoors or partially outdoors. Secondly, we have determined several brain molecular markers, serum catecholamines and serum biochemical parameters related to physical damage and stress. The goal is to understand the neurological response to different management conditions and to ascertain whether pigs living partially outdoors cope differently with road transport stress than pigs in intensive conditions.

## Materials and methods

### Experimental design

In this study, 24 female pigs ((Landrace x Large White) x Piétrain) were used. At 9 weeks of age, pigs were transferred from a commercial farm to the experimental facilities of IRTA (Institut de Recerca i Tecnologia Agroalimentàries, Veinat de Sies s/n, Monells, Spain) and housed in 4 pens of 6 pigs each. Pens (5 x 2.7 m) had fully slatted floor and were under natural light conditions at a constant environmental temperature (22 ± 3°C). Each pen was provided with one steel drinker bowl (15 x 16 cm) connected to a nipple and a concrete feeder (58 x 34 cm) with four feeding places. Pigs had water and food ad libitum. Pigs were inspected daily and no health problems were observed during the experimental period. At 22 weeks of age, two groups remained in the conditions previously described (indoor-group, ID group, n = 12, 6 for each pen). The other two groups (outdoor-group, OD group, n = 12, 6 for each pen) was housed combining indoor conditions with 4 hours (from 9:00 until 13:00) of outdoor pasture. The outdoor pasture consisted in 250 m^2^ of unpaved ground with mud and grass surrounded by a fence, with covered area to protect pigs from sun and access to a drinking point.

At 26 weeks of age, pigs were transported to the IRTA’s experimental slaughterhouse (Finca Camps i Armet, Monells, Spain). One set of animals (n = 12) from each housing condition (ID/OD, n = 6 for each condition) was managed in low-stress conditions (LS) consisting in keeping the same groups in the truck than in the farm, soft management during loading/unloading the truck, soft drive for 5 min to the slaughterhouse 1.2 km far away (low-stress group, LT). Thus, pigs were always managed without mixing.

The remaining animals (n = 12, 6 of each ID/OD condition) were managed in high stress conditions (HS) consisting in transporting the 12 animals mixed in the truck, harsh management during loading and unloading (loud voices, rough handling), 2 hours drive by a winding road (high-stress group, HS group). On arrival, pigs were immediately moved in groups of 3 to a gas stunning system and exposed to 90% CO_2_ for 3 min before exsanguination.

The study was approved by the Institutional Animal Care and Use Committee (IACUC) of IRTA.

### Blood and brain sampling

Blood samples were collected at exsanguination from each pig in 10-mL tubes without anticoagulant. Blood was allowed to clot and serum was obtained by centrifugation at 2000 x g for 10 min and it was frozen at -80°C until analysis.

Immediately, 5 min maximum after the slaughter, the skull was opened. The brain was removed and the HC and PFC were excised, collected as quickly as possible (90 s maximum) in liquid N2 and kept frozen at -80°C.

### Serum biochemistry

Haptoglobin (Hp) was determined spectrophotometrically (Phase Haptoglobin, Tridelta Ltd, County Kildare, Ireland). Creatine kinase (CK) was determined with the IFCC (International Federation of Clinical Chemistry) method (Olympus System Reagent OSR# 6179). Both techniques were adapted to the Olympus AU400 analyser (Beckman Coulter, Ireland). Pig-MAP was measured by ELISA (PigChamp ProEuropa, Segovia, Spain). Cortisol concentrations were determined by ELISA (DRG Cortisol ELISA, DRG Diagnostics, Marburg, Germany).

### Brain extracts preparation

Brain samples (PFC and HC) were weighted and homogenized in ice-cold 0.15 M NaCl, 0.05 M Tris-HCl pH 8.0 and 1.0% Triton X-100 buffer with protease inhibitors (protease inhibitors cocktail, Sigma-Aldrich, St. Louis, MO) and dihydroxybenzylamine (DHBA) as internal standard (0.3 g of tissue/mL). The mixtures were homogenized by sonication (Branson Digital Sonifier, model 250, Branson Ultrasonics Corp., Danbury, CT) and the brain extracts were kept frozen in aliquots at -80°C.

### Monoamine neurotransmitter quantification

Brain extracts were homogenized (1:2) in ice-cold 0.25 M perchloric acid containing 0.1 M NaS_2_O_5_ and 0.25 M ethylenediaminetetraacetate (EDTA) and kept frozen. After centrifugation at 12000 x g for 10 min at 4°C, the concentration of catecholamines (NA, DA, DOPAC and HVA) and indoleamines (5-HT and 5-HIAA) were determined in 20 μL aliquots using HPLC (Elite LaCHrom, Merck, Hitachi, Japan) equipped with a Chromolith Rp-18e 100 x 4.6 mm column (Merck KgaA, Darmstadt, Germany) with electrochemical detection (ESA Coulochem II 5200, Bedford, MA). The mobile phase consisted of 0.5 M citrate buffer pH 2.8, 0.05 mM EDTA, 1.2 mM sodium octyl sulphate (SOS) and 1% acetonitrile. The applied voltage was set at 400 mV and the flow rate was 1 mL/min [50]. Validation of the methodology is described in Arroyo et al. [49]. The internal control DHBA allowed the comparison between runs. Dopaminergic total system (DA-system) and serotonergic total system (5-HT-system) are calculated as the sum of all metabolites in the pathway (DA, DOPAC and DA; and 5-HT and 5-HIAA; respectively). Noradrenergic system (NA-system) is only composed by NA concentration.

### Serum catecholamines

Serum catecholamines were extracted following the procedure described by Caroldi [51]. Approximately 10 mg of activated and acid washed alumina was added to 0.1 mL of serum with DHBA as an internal standard, followed by 0.5 ml of 1.5 M Tris buffer (pH 8.6 containing 0.07 M EDTA). Samples were shaken vigorously for 30 min, the alumina was precipitated by centrifugation at 300 x g for 1 minute, washed by centrifugation two times with 1 mL of water. Catecholamines were eluted from the alumina by the addition of 100 μL of 0.25 M perchloric acid containing 0.1 M NaS_2_O_5_ and 0.25 M EDTA and centrifugation at 300 x g for 1 minute. NA and A concentrations were determined in 20 μL aliquots using HPLC as described above.

### Immunoblotting

Antibodies: anti-c-Fos and anti-β-actin antibodies were from Santa Cruz Biotechnology (Santa Cruz, CA, USA), anti-ERK1/2 was from Cell Signaling (Danvers, MA, USA) and Anti-dinitrophenyl (DNP) was from Sigma (St. Louis, MO, USA). Goat anti-rabbit IgG (H+L) HRP-linked antibody was from Cell Signaling (Danvers, MA, USA) and sheep anti-mouse IgG HRP-linked whole antibody was from GE Healthcare (Buckinghamshire, UK).

Total protein concentration of brain samples was determined using the colorimetric Bio-Rad protein assay, based on the Bradford dye-binding method, according to kit instructions (Bio-Rad, München, Germany).

For Western Blot, 40 μg of protein from HC and PFC extracts were resolved under reducing conditions in SDS-PAGE (12% acrylamide) and transferred onto polyvinylidene fluoride (PVDF) membranes (Immun-Blot PDVF, Bio-Rad, Hercules, CA, USA) for 45 min at 300 mA in a semidry transferring system. Since several gels were run to process all samples, a unique brain sample was resolved in all SDS- PAGE as internal control to allow inter-gel comparison. Membranes were then blocked for 1 h at room temperature with 5% fat-free milk in TBS-0.05% Tween-20 (TBS-T). Membranes were incubated with the diluted antibodies (1/200 for c-Fos detection and 1/2000 for ERK1/2, 1/10000 for β-actin) for 16 h at 4°C (1 h at room temperature for β-actin), washed and further probed with horseradish peroxidise (HRP)-conjugated immunoglobulin for 1 h at room temperature. Antibody binding was visualized by chemiluminescence (ECL, GE Healthcare, Buckinghamshire, UK). Densitometry was performed in a LAS-3000 Luminiscent Image Analyzer with the Multi Gauge software (Fujifilm Corporation, Tokyo, Japan). Standardization was performed against β-actin densitometry to correct for the protein content of the samples and against a control brain sample to correct inter-gel variation.

For protein carbonylation content (PC) detection, a Slot Blot was performed, as described in [52] with some modifications. Lysates from HC and PFC (5 μg) were applied to each slot. After an incubation of 20 min, vacuum was applied until all the liquid above the PVDF membrane disappeared. Sequentially, the membrane was incubated for 1 min in MeOH, in Transfer Buffer (0.25 M Tris, 0.19 M glycine and 20% MeOH) and then in 2 N HCl. Samples were derivatized by incubating the membrane in a solution of 2,4-dinitrophenylhydrazine (DNPH, 100 mg/mL) in 2 N HCl for exactly 5 min. Sequentially, the membrane was washed for 5 min twice in 100% MeOH, twice in Transfer Buffer and then twice in TBS-T. Membranes were incubated overnight at 4°C with the primary antibody (diluted 1/25000 for DNP and 1/10000 for β-actin) and washed and further probed with horseradish peroxidise (HRP)-conjugated immunoglobulin for 1 h at room temperature. Antibody binding was visualized by chemiluminescence (ECL, GE Healthcare, Buckinghamshire, UK). Densitometry was performed in a LAS-3000 Luminiscent Image Analyzer with the Multi Gauge software (Fujifilm Corporation, Tokyo, Japan). Standardization was performed against β-actin densitometry to correct for the protein content of the samples. Samples were assayed by duplicate in two different membranes.

### BDNF determination in the HC

HC extracts were diluted 1:5 with DPBS (2.68 mM KCl, 137 mM NaCl, 1.47 mM KH_2_PO_4_, 8.1 mM Na_2_HPO_4_, 0.9 mM CaCl_2_ 2H_2_O and 0.49 mM MgCl_2_ 6H_2_O) and analyzed by ELISA according to the manufacturer’s instructions (Promega, Madison, WI). BDNF concentrations were interpolated from the standard curve (pg/mL) and calculated as pg of BDNF/mg of protein.

### Statistical analysis

All statistical analysis was performed in SPSS 22.0 software (IBM, Chicago, IL, USA). The significance level was established at P < 0.05 and a tendency was considered at 0.05 ≤ P ≤ 0.1. Descriptive data are presented with the means and the standard error (mean ± SE).

#### Univariate analysis

Whenever possible, data were log transformed to correct the distribution and hence permit use of parametric statistics. Normality test of data and residuals was performed for each measure. Normally distributed measures were analyzed using the UNIANOVA procedure of SPSS with Tukey adjustment. HVA in the PFC did not show normal distribution and no parametric GENLIN procedure was performed. In all models, each pig was introduced as the experimental unit, the fixed effects included were stress (low (LS) and high (HS), housing conditions (outdoor (OD) and indoor (ID)) and their interaction). When required, pairwise comparison with Bonferroni adjustment was performed.

Correlations between variables were analysed with Pearson procedure for normal distributed variables and Spearman procedure for HVA in the PFC. Since NTs in the same pathway were always highly correlated in each region, only dopaminergic total system (DA-system) and serotonergic total system (5-HT-system) were used in the correlation analysis.

#### Data preprocessing and multivariate analysis

NA in the PFC had > 20% of missing data and was therefore removed from further analysis. For the other variables, all missing data including removed outliers and missing samples were imputed using the SPSS multiple imputation package.

Differences between ODHS, IDHS, ODLS and IDLS pigs were investigated applying the multivariate approach Fisher Discriminant Analysis. Initially, 22 variables (serum Pig-MAP, Hp, CK, NA, A and cortisol; HC c-Fos, ERK1/2, BDNF, PC, NA, DA, total DA-system, 5-HT and total 5-HT-system; PFC c-Fos, ERK1/2, PC, DA, total DA-system, 5-HT, total 5-HT-system) were included in the Discriminant Analysis model, and then, the stepwise method selected the discriminant variables on basis of Wilks’ lambda statistic. The F value was set at Fentry = 3.84 and Fremoval = 2.71. Only functions with eigenvalues > 1 were selected. Internal cross-validation using leave-one-out method was performed to measure the robustness and accuracy of the discriminant analysis. A discriminant score was assigned to each subject for each discriminant function.

## Results

### Serum biochemistry and catecholamines

Results are presented in Table 1 and S1 Table. There was an effect of housing for the acute phase protein Hp (higher in ID pigs, *P* = 0.003) and the same tendency was observed for another acute phase protein, Pig-MAP (*P* = 0.066). In this case there was also an effect of transport stress (*P* = 0.006) and an interaction of both factors (*P* = 0.035), indicating an effect of stress in ID pigs and an effect of housing in LS pigs. CK, a widely used marker for skeletal muscle damage, was increased by HS (*P* < 0.001), whereas no effect of housing nor interaction between both factors was observed. Cortisol, NA and A did not show differences by any of both factors. An interaction of transport and housing was observed for NA serum concentration (*P* < 0.001).

**Table 1:** Serum CK, Hp, Pig-MAP, catecholamines (NA and A) and cortisol in pigs raised indoors (ID) or partially outdoors (OD) and submitted to low (LS) or high (HS) transport stress. Data are presented as means and SE. *P* values from univariate statistical analysis including Housing, Transport Stress and their interaction Housing*Stress (H*S) are shown. Statistical significant *P* values are in bold.

Parameter	Housing Condition	Transport		Statistics (P values)		
		LS	HS	*Housing*	*Stress*	H*S
CK (U/mL)	*ID*	1.97 ± 0.2	7.78 ± 2.04	0.096	***<0*.*001***	0.294
	*OD*	2.37 ± 0.38	12.08 ± 1.74			
Hp (mg/mL)	*ID*	0.4 ± 0.07	0.34 ± 0.03	***0*.*003***	0.149	0.703
	*OD*	0.23 ± 0.07	0.13 ± 0.04			
Pig-MAP (mg/mL)	*ID*	0.93 ± 0.06 ^aA^	0.63 ± 0.04 ^b^	0.066	***0*.*006***	***0*.*035***
	*OD*	0.69 ± 0.08 ^B^	0.65 ± 0.04			
NA (ng/mL)	*ID*	472.36 ± 32.21 ^aA^	347.03 ± 28.48 ^Ab^	0.863	0.149	***<0*.*001***
	*OD*	289.88 ± 48.00 ^aB^	517.64 ± 25.61 ^bB^			
A (ng/mL)	*ID*	131.77 ± 17.21	124.57 ± 22.11	0.377	0.394	0.227
	*OD*	90.56 ± 26.14	131.13 ± 9.30			
Cortisol (ng/mL)	*ID*	43.70 ± 2.67	34.43 ± 5.35	0.901	0.702	0.234
	*OD*	35.94 ± 7.15	40.75 ± 5.85			

Different lower case superscripts indicate different means in the same row *(P <0*.*05)*. Different capital letter superscripts indicate different means in the same column *(P <0*.*05)*.

### Brain monoamine NT profiles in PFC and HC

The concentrations of brain monoamines and their metabolites in PFC and HC are presented in Table 2 and S1 Table.

**Table 2:** Monoamine neurotransmitter profile in PFC and HC in pigs raised indoors (ID) or partially outdoors (OD) and submitted to low (LS) or high (HS) transport stress. DA and 5-HT systems represent the sum of total metabolites in each pathway (DA, DOPAC and HVA; and 5-HT and 5-HIAA, respectively). Data are presented as means and SE. *P* values from univariate statistical analysis including Housing (H), Transport Stress (S) and their interaction Housing*Transport Stress (H*S) is shown. Statistical significant *P* values are in bold.

Sample	Parameter (ng/mg of tissue)	Housing Condition	Transport		*Statistics (P values)*		
			LS	HS	*Housing*	*Stress*	H*S
*PFC*	NA	*ID*	97.92 ± 11.12	196.73 ± 20.67	0.828	***<0*.*001***	0.657
		*OD*	112.83 ± 27.95	191.60 ± 5.60			
	DA	*ID*	65.74 ± 10.35	173.64 ± 40.36	***0*.*001***	***0*.*001***	0.830
		*OD*	165.72 ± 28.65	413.17 ± 82.06			
	DOPAC	*ID*	ND	ND	ND	***ND***	ND
		*OD*	ND	ND			
	HVA	*ID*	20.55 ± 11.95	59.50 ± 26.68	0.190	***0*.*008***	0.827
		*OD*	45.14 ± 21.15	241.42 ± 88.41			
	DA-sytem	*ID*	75.29 ± 10.81	233.13 ± 40.38	***<0*.*001***	***<0*.*001***	0.727
		*OD*	210.82 ± 47.27	654.58 ± 64.92			
	5-HT	*ID*	167.64 ± 18.01	140.47 ± 17.84	0.576	***0*.*046***	0.757
		*OD*	163.85 ± 10.71	127.36 ± 12.23			
	5-HIAA	*ID*	79.36 ± 5.77	70.28 ± 5.17	***0*.*002***	0.571	0.389
		*OD*	96.03 ± 7.20	97.93 ± 6.11			
	5-HT-system	*ID*	246.99 ± 23.30	210.75 ± 21.43	0.494	0.087	0.967
		*OD*	259.87 ± 17.24	225.29 ± 15.84			
*HC*	NA	*ID*	256.96 ± 30.01	296.6 ± 56.87	1.000	1.000	1.000
		*OD*	243.6 ± 31.32	328.44 ± 34.48			
	DA	*ID*	110.32 ± 13.99	172.02 ± 51.33	0.482	***0*.*005***	***0*.*043***
		*OD*	85.36 ± 15.27 ^a^	250.37 ± 21.49 ^b^			
	DOPAC	*ID*	52.53 ± 2.80	60.68 ± 5.05	0.978	***<0*.*001***	0.065
		*OD*	45.80 ± 2.57	67.22 ± 1.59			
	HVA	*ID*	397.66 ± 26.75	553.35 ± 49.89	0.584	***<0*.*001***	0.356
		*OD*	383.56 ± 28.49	607.88 ± 35.39			
	DA-sytem	*ID*	560.50 ± 33.80 ^a^	709.84 ± 50.88 ^Ab^	0.057	***<0*.*001***	***0*.*006***
		*OD*	514.72 ± 36.76 ^a^	928.75 ± 48.69 ^Bb^			
	5-HT	*ID*	208.81 ± 5.30	234.88 ± 14.40	1.000	1.000	1.000
		*OD*	238.71 ± 11.12	188.90 ± 19.14			
	5-HIAA	*ID*	52.56 ± 3.86	62.42 ± 4.00	***0*.*037***	***0*.*014***	0.799
		*OD*	42.43 ± 2.73	54.38 ± 5.25			
	5-HT-system	*ID*	259.93 ± 5.71	297.31 ± 16.45	1.000	1.000	1.000
		*OD*	280.78 ± 10.40	243.28 ± 21.70			

Different lower case superscripts indicate different means in the same row *(P <0*.*05)*. Different capital letter superscripts indicate different means in the same column *(P <0*.*05)*.

In the PFC, living indoors or partially outdoors does have a significant effect on DA concentration, being higher in OD animals (*P* = 0.001), leading to an increase in total DA-system (*P* < 0.001). Levels of 5-HIAA are also affected by housing conditions (*P* = 0.002), showing OD animals higher levels of the 5-HT catabolite.

The PFC was extremely sensitive to transport stress, being NA, and the DA and 5-HT pathways altered. The PFC showed an elevated catecholamine pathway with higher concentration of NA (*P* < 0.001), and DA (*P* = 0.001) and HVA (*P* = 0.008) with a concomitant important increase in DA-system in HS pigs (*P* < 0.001). In contrast, the concentration of 5-HT decreased slightly (*P* = 0.046).

In the HC, DA and its metabolites DOPAC and HVA were increased in HS group (*P* < 0.001 for DOPAC and HVA, and *P* = 0.005 for DA), and as a consequence total DA-system was increased as well (*P <* 0.001). An interaction between both factors was observed for DA (*P* = 0.043) and Bonferroni analysis indicated that the effect of stress was stronger in OD pigs. For indoleamines, a slight decrease in 5-HIAA concentration is observed in OD pigs versus ID pigs (*P* = 0.037). A slight increase in 5-HIAA was observed in HS group compared to LS group (*P* = 0.014).

### Molecular markers of stress in PFC and HC: c-Fos, ERK1/2 and protein carbonylation (PC)

**Fig 1:**
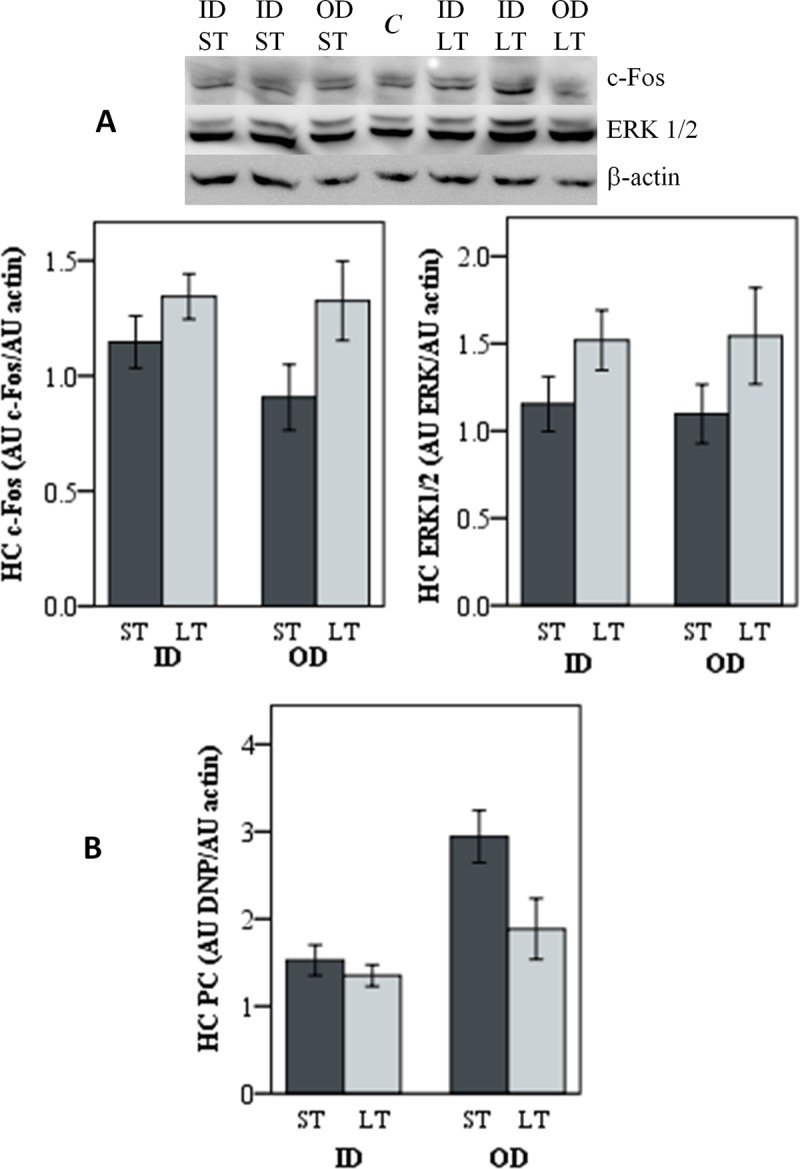
Molecular markers in the HC of pigs raised indoors (ID) or outdoors (OD) and transported to the slaughterhouse in low stress (LS) or in high stress (HS) conditions. (A) Representative Western blot of c-Fos and ERK1/2 and densitometry of all individual samples (n = 24). A sample brain was used as internal control (C) to allow inter-gel comparison. Actin was used as loading control. Results are presented as ratios of absorbance units (AU) of protein band to AU of β-actin. (B) Protein carbonylation (PC) represented as AU of DNP groups to AU of β-actin ratio. Data is shown as mean ± SE.

In the HC, the level of c-Fos and ERK1/2 was similar in animals in both housing conditions (ID, OD) (Table 3 and S1 Table). Nevertheless, an increase in the intensity of the band corresponding to these proteins was observed after high stress transport conditions for both proteins (Fig 1A). Statistical analysis showed an effect of transport for c-Fos (*P* = 0.036) and a tendency for ERK1/2 (*P* = 0.055). The level of carbonyl groups in proteins (PC) was affected by housing conditions (lower levels in ID pigs, *P* = 0.001) and transport stress (lower in HS pigs, *P* = 0.025) (Fig 1B). No significant effects were observed for any of these parameters in the PFC (Table 3).

**Table 3:** Brain molecular and oxidative stress markers in PFC and HC in pigs raised indoors (ID) or partially outdoors (OD) and submitted to low (LS) or high (HS) transport stress. Results are presented as ratios of absorbance units (AU) of protein band to AU of β-actin (mean ± SE). *P* values from univariate statistical analysis including Housing (H), Transport Stress (S) and their interaction Housing*Transport Stress (H*S) is shown. Statistical significant *P* values are in bold.

Sample	Parameter	Housing Condition	Transport		*Statistics (P values)*		
			LS	HS	*Housing*	*Stress*	H*S
*PFC*	**PC**	*ID*	0.85 ± 0.09	0.60 ± 0.07	0.222	0.143	0.454
		*OD*	0.62 ± 0.09	0.54 ± 0.10			
	**c-Fos**	*ID*	0.75 ± 0.21	0.76 ± 0.12	0.306	0.899	0.948
		*OD*	0.99 ± 0.28	1.03 ± 0.15			
	**ERK1/2**	*ID*	0.56 ± 0.15	0.58 ± 0.19	0.693	0.400	0.458
		*OD*	0.50 ± 0.08	0.79 ± 0.22			
*HC*	**PC**	*ID*	1.53 ± 0.75	1.35 ± 0.13	***0*.*001***	***0*.*025***	0.097
		*OD*	2.94 ± 0.30	1.89 ± 0.35			
	**c-Fos**	*ID*	1.15 ± 0.11	1.34 ± 0.10	0.351	***0*.*036***	0.424
		*OD*	0.91 ± 0.14	1.33 ± 0.17			
	**ERK1/2**	*ID*	1.15 ± 0.16	1.52 ± 0.17	0.933	0.055	0.842
		*OD*	1.10 ± 0.17	1.54 ± 0.28			

### Hippocampal BDNF

A decrease on the HC concentration of the neurotrophic factor BDNF was observed in HS animals (Table 4 and S1 Table, *P* = 0.012). No effect of housing and no interaction between both factors were shown.

**Table 4:** BDNF in HC in pigs raised indoors (ID) or partially outdoors (OD) and submitted to short (ST) or long (LT) road transport. Data are presented as means and SE. *P* values from univariate statistical analysis including Housing, Transport Stress and their interaction Housing*Stress (H*S) are shown. Statistical significant *P* values are in bold.

Parameter	Housing Condition	Transport		*Statistics (P values)*		
		LS	HS	*Housing*	*Stress*	H*S
**BDNF** (pg/mg protein)	*ID*	22.53 ± 1.84	17.31 ± 2.36	0.804	***0*.*012***	0.397
	*OD*	24.20 ± 3.74	14.27 ± 2.24			

Raw data is accessible at S1 Table.

### Multivariate analysis

**Fig 2:**
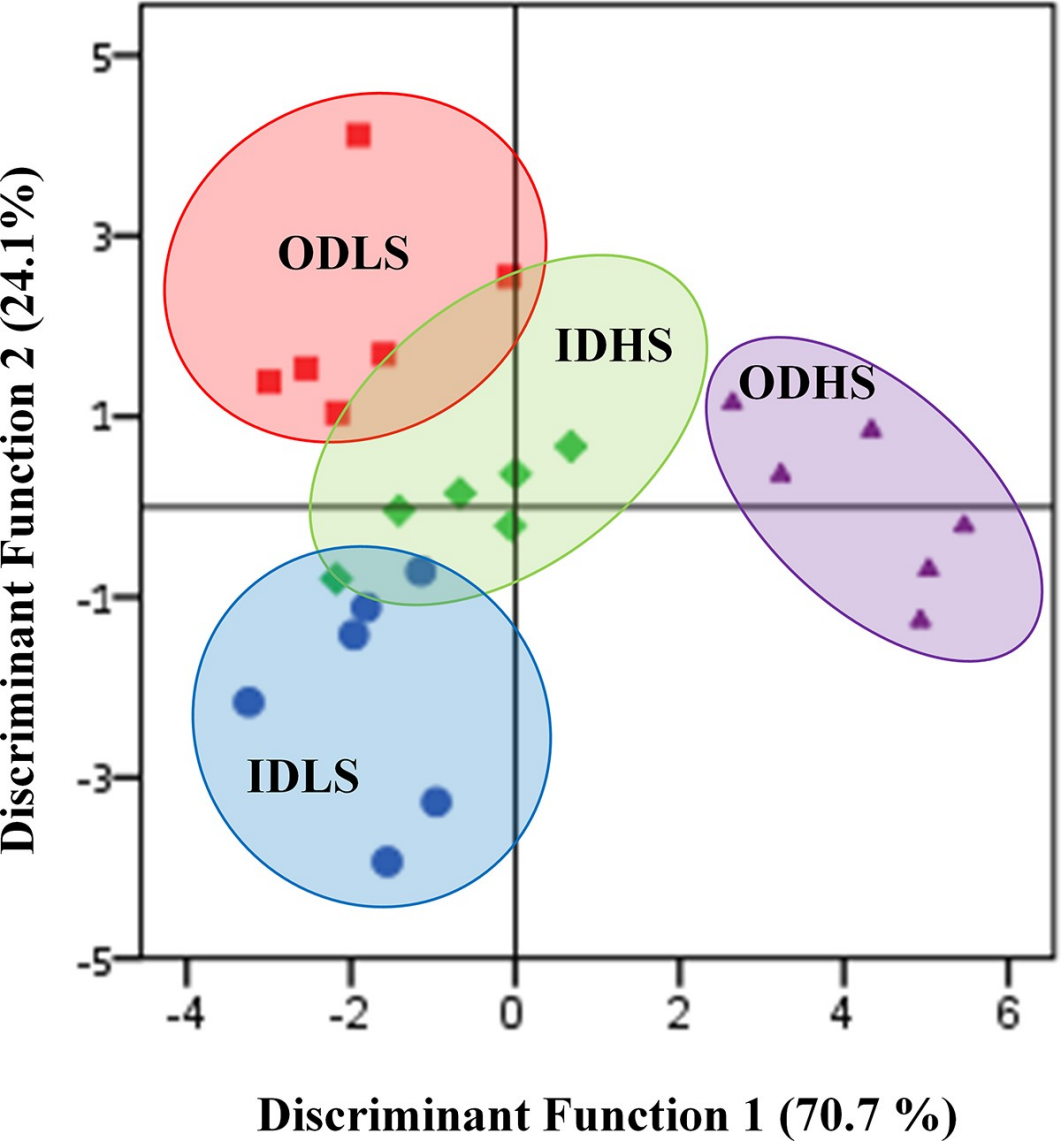
Score plot from a discriminant analysis. Each subject is represented according the score obtained for discriminant functions 1 and 2. The colour of these points and of the grouped oval indicates the experimental groups (IDLS, IDHS, ODLS and ODHS).

Stepwise discriminant analysis of 22 variables of HC, PFC and serum samples selected 4 variables and 3 discriminant functions that were significantly associated with housing and stress treatments: DA-system in the PFC, NA in serum, PC in HC and 5-HT in HC. The inclusion of other parameters did not improve the discrimination ability of the test. The Wilk’s Lambda discriminant analysis indicated that there was low overlapping of confident ellipse of IDHS-group with ODLS and IDLS groups, except for one IDLS and two ODLS pigs that were included in IDHS group, and one IDHS that was included in IDLS group (Fig 2). The self-verification using internal cross-variation (leaving-one-out method) revealed high accuracy (83.3%) with only 4 variables (Table 5). Details on the Wilk’s Lambda discriminant analysis are shown in S2 Table.

**Table 5:** Classification results of self-verification of the discriminant analysis.

Actual classification	Predicted group membership			
	IDLS (%)	IDHS (%)	ODLS (%)	ODHS (%)
IDLS	5 (83.3)	1 (16.7)	0	0
IDHS	1 (16.7)	5 (83.3)	0	0
ODLS	0	2 (33.3)	4 (66.7)	0
ODHS	0	0	0	6 (100)

There were 21 individuals correctly classified (83.3%)

### Correlation between all parameters

**Fig 3:**
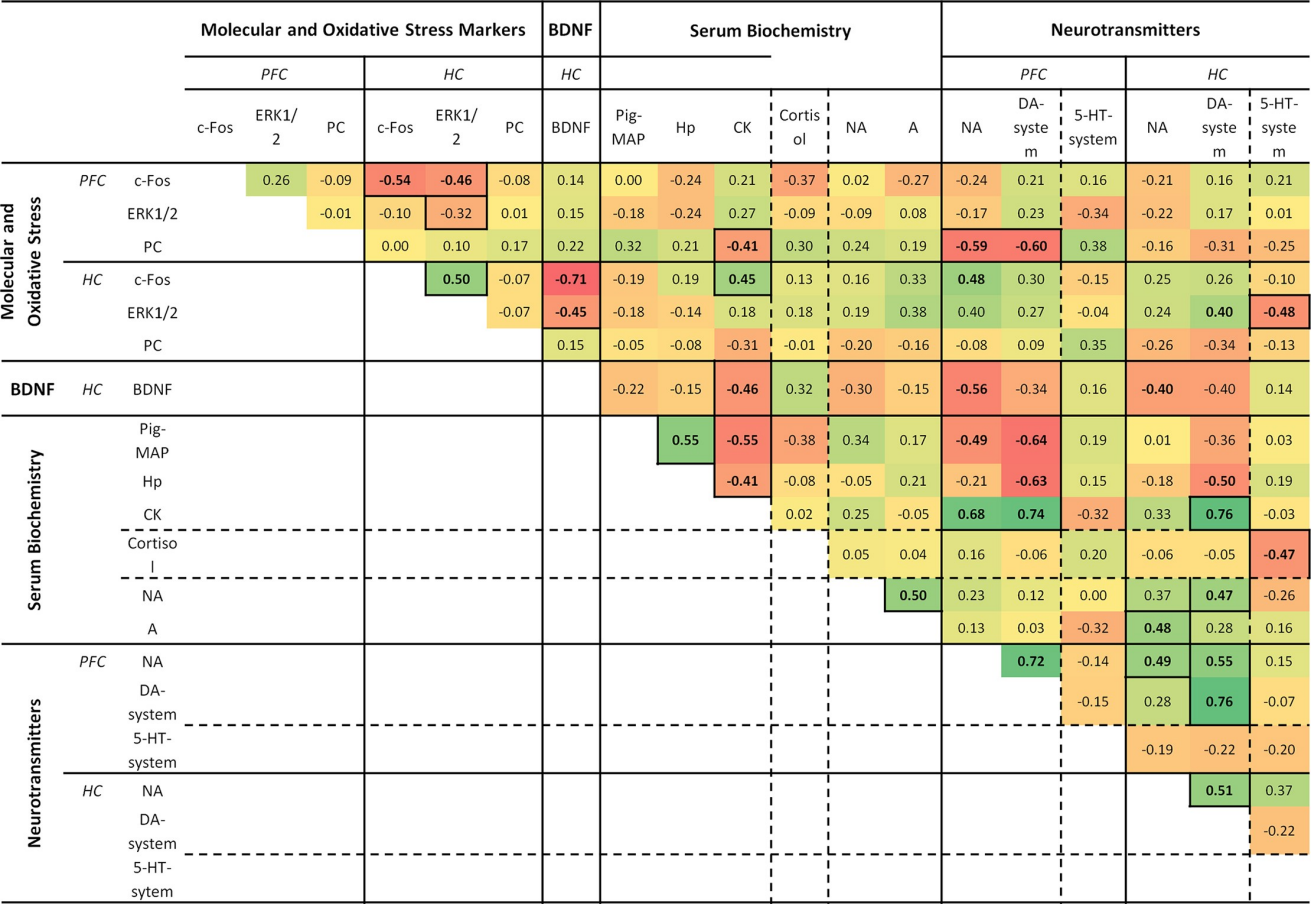
Correlation coefficients of all measured parameters. Bold numbers indicates significant correlations (P < 0.05). Positive correlations are in green and negative correlations are in red. Colour degradation represents the strength of the correlations from R = 1 to R = -1.

Correlations among all the variables are presented in Fig 3. Since NTs in the same pathway were always highly correlated in each region (i.e. DA, DOPAC, HVA; 5-HT, 5-HIAA), the terms “DA-system” and “5-HT-system” are used for simplicity. Only correlations with R>0.5 and *P*< 0.05 were considered for discussion.

*Positive correlations*: In the PFC and in the HC, the DA-system is correlated to NA within the same area. Both systems are also correlated between areas (DA-system in PFC *vs* HC, and NA-system in PFC *vs* HC). The DA-system in the HC is also correlated to NA in the PFC. Both the DA and NA -systems in the HC are also correlated to serum catecholamine concentration. c-Fos is correlated to ERK1/2 in the HC. Serum CK is correlated to the DA and NA -systems in the PFC and to the DA-system in the HC.

*Negative correlations*: BDNF is correlated c-Fos in the HC and with NA in the PFC. The NA and DA -systems are correlated to PC in the PFC, and to serum acute phase proteins (Hp and Pig-MAP). Serum CK is correlated to serum acute phase proteins, specially Pig-MAP. c-Fos in the PFC is correlated to c-Fos and ERK1/2 in the HC.

## Discussion

The main objective of the present work was to assess the molecular changes in the HC and the PFC in pigs after combining two management factors: housing conditions and transport-associated stress. In this experiment animals housed during all day indoors (ID), which is the normal condition in intensive production systems, were compared to animals spending 4 hours per day outdoors (OD) in a large area during the last month before slaughter. Animals from both groups were then subjected to two types of road transport: low-stress conditions (LS) consisted in keeping the same pig groups, soft handling procedures and 5 min quiet driving, whereas high-stress conditions (HS) consisted in mixing groups, harsh handling procedures and 2 hours in a truck driving by uncomfortable roads. Our hypothesis was that animals housed partially outdoors would cope better with the stress conditions of the transport than those animals kept always indoors.

### Does living partially outdoors modify serum biochemistry and the brain neurotransmitter systems?

Outdoors systems have been claimed to provide the animals with an EE [3,4]. Indeed, our results indicate that pigs raised in this environment four hours per day had lower serum levels of the acute phase proteins Hp and Pig-MAP (tendency). These proteins are well-accepted markers for infection or inflammation, but they are also indicators of stress [53–56], suggesting that outdoors rearing contribute to a better welfare. Although it may be expected that serum cortisol would also be lower in OD pigs, and in accordance with previous studies investigating plasma or salivary cortisol [9,57–59], serum cortisol levels were not affected by the rearing system. It has to be taken into account that blood samples were taken at exsanguination, and the acute stress due to the sacrifice should be similar for all the individuals, masking the potential cortisol differences due to housing.

Many studies in rodents have shown that EE modifies the profile of NT in the CNS [31,60]. In pigs, changes in neuronal morphology in young pigs subjected to EE have been reported [61] but the effects on brain neurochemistry have not been analyzed. The current study is the first one to demonstrate changes in NT pathways and EE in the fattening pig. The main differences were observed in the DA system in the PFC, with an important increase in DA and total DA-system, similar to the higher PFC dopaminergic neurotransmission described in rats subjected to EE [34]. DA is known for its main role in reward-seeking behaviour, since DA in the brain is linked to feelings of pleasure and general well-being [62,63]. Hence, our results support the good consequences of outdoor housing in pig production. Our results would also support a relationship of the serotoninergic system to EE, since HIAA, the metabolite of 5-HT, was slightly but significantly increased in the PFC and slightly decreased in the HC in OD pigs. The relationship of serotonin to EE is a controversial matter since data in the literature are sparse [36].

Surprisingly, there were no changes associated to housing in BDNF in the HC, although it has been reported that EE enhances the levels of BDNF[31,37,38]. BDNF is a neurotrophin that plays an important role in synaptic plasticity and neuronal survival [39,40] and hippocampal BDNF has been mainly related to learning and memory formation [64]. It is possible that OD housing did not provide a sufficiently enrichment for pigs, the time of housing (4 weeks) was too short or the large interindividual variation was masking the concentration changes. Another possibility is that changes in BDNF have been associated to local variation associated to the dorsal HC, but not the ventral HC [65]. Since the whole HC was used in the present work this may be again due to low sensitivity. A high interindividual variation in BDNF concentration has been described in pigs and other species [66].

### Does transport-associated stress modify serum biochemistry and the brain neurotransmitter systems?

In serum biochemical variables, an increase in CK concentration was observed in the HS group indicating the existence of muscular damage probably associated to a higher proportion of injuries due to transport and mixing.

In the CNS, several reports have shown the relationship between monoaminergic pathways and conditions as stress and aggression [43,44,46–49]. In our study, significant changes in the catecholaminergic systems (DA and NA) were observed. Transport-associated stress largely activated the DA pathway in the PFC and in the HC. Although, as stated above, DA has been associated to reward pathways, neurochemical studies have demonstrated that the DA system is activated by stressful stimuli [14,22,67]. This stress-induced hyperdopaminergic state is driven by the HC in a way that, if a threat or specific situation requiring high vigilance is present, the HC will set the DA system to a higher level of activity, rendering it more reactive to deliver the appropriate response or facilitating escape. The mechanism may be driven by glucocorticoids (GC) [68]. The evolutive meaning is stress being helpful for memory consolidation, which is mediated by the HC [69]. Memories are then transferred to the cortex, important for the executive, cognitive and emotional control of behaviour [13]. Indeed, our results show that the DA pathway was also activated in the PFC in HS conditions. Likewise, changes in DA in the PFC during stress have been associated to a state of vigilance, leading to a better control of locomotion and increasing neuronal information processing [70]. In laboratory animals, exposure to a variety of stressors results in an increase of DA as measured by postmortem neurochemistry [71]. This coincidence of DA being associated to well-being as well as stressful situations is probably linked to the highly polemic discussion about DA’s role in pleasure and pain [72].

An increase in NA was observed in the PFC after transport-associated stress. The PFC is a crucial component in the responses to stressful stimuli and it has been suggested that it is selectively activated by social or psychological stressors [68], which should be the case in pigs after group mixing and transport in a truck. Previous studies in laboratory animals have shown that both NA turnover and release increase with stress in brain regions mediating stress responses such as the cortex [73], similar to the situation observed in the present study. Similar to DA, the noradrenergic signalling has been linked to HPA axis responses [73].

The serotoninergic system was mildly affected by HS conditions since 5-HT was decreased in the PFC and 5-HIAA was increased in the HC. This is in agreement with results described in pigs, where it has been shown that 5-HT release is increased in the HC in several stress conditions, mostly acute, and influenced by individual characteristics [49].

A general marker of neuronal activation is the induction of the early-response gene c-*FOS*. It is generally accepted that c-Fos induction reflects the functional activity of neurons in stress-related neuronal circuitries and it occurs in a wide range of brain structures after exposure to various acute stressors, including the HC [23,25,26], whereas the ERK pathway has been proposed to act upstream of c-Fos induction [29]. In our experimental design, exposure to HS conditions induced higher levels of c-Fos expression and a tendency for ERK1/2 in the HC. These effects were observed after road transport for 2h, in agreement with the reports about c-Fos expression occurring within a few minutes with maximal levels at 60–90 min after the stressor [25,27]. In pigs, *c-FOS* mRNA concentration was increased in piglets born from sows subjected to reallocation during late pregnancy, indicating a greater neuronal activation of the HC and increased perception of a stressful situation [45].

In contrast to housing, hippocampal BDNF was decreased to a 60% after road transport-associated stress. Indeed, many different types of acute and chronic stressors decrease BDNF expression in the HC and low hippocampal BDNF expression is consistently associated with depressive-like behaviour [39,40,67].

### Correlation between parameters

c-Fos and ERK1/2 appeared to be highly correlated in the HC (R = 0.629, *P* = 0.002). c-Fos was also correlated to the DA-system in the HC (R = 0.503 and *P* = 0.020) and NA in the PFC (R = 0.541 and *P* = 0.030). Globally, there was a positive correlation between the DA and NA systems in both brain areas, and also to serum catecholamines. Altogether, these relationships confirm the mechanisms linking neuronal activation, signal transduction and the induction of NT central pathways during stress [74,75].

Interestingly, serum CK was highly correlated to these NT systems. Furthermore, BDNF was negatively correlated to stress-linked brain NT pathways and to serum CK. In this way, stress-associated neurochemical pathways were linked to physical muscular damage.

### Interaction between housing and transport

In the overall, our results indicate that animals living outdoors respond differently to transport-associated stress than animals living indoors. This is suggested by the interaction between housing and transport in serum NA concentration, a marker of acute stress, and also an interaction between housing and transport in the DA-system in the HC.

The discriminant analysis also supported this conclusion. Animals raised outdoors and submitted to a high transport-associated stress (Fig 2, ODHS group, violet oval) were clearly differentiated from the other groups. Animals living indoors and outdoors subjected to a short and mild transport (IDLS and ODLS groups, blue and red oval, respectively) were also clearly discriminated, indicating that even a relatively short time in the open air clearly has an effect on the physiology of these animals.

### Welfare implications

One main implication of our work is that living outdoors, even during some hours per day, has beneficial effects as visualized by changes on serum stress markers as well as in the HC dopamine system.

Another important question is whether EE modifies the behavioural and the physiological responses to stressors, as suggested by previous studies performed in growing or fattening pigs [4]. Several authors found that pigs reared outdoors show less aggressive interactions after mixing at loading, during transport and lairage than conventionally reared pigs, even with the same level of mixing, and it has been suggested that pigs from EE deal more adequately with transport and pre-slaughter handling [3,5,59,76]. Furthermore, pigs that are used to handling and exercise on a farm, like those moved every day to outdoors, display a calmer behaviour and are more fit, resulting in better handling during transport [77]. Recently, Rocha et al. [78] reported a greater reluctance to move at loading and greater percentage of turning back and slips at unloading in pigs raised at conventional farms compared with pigs raised through animal welfare improved raising system (higher space allowance, presence of bedding, quiet handling and frequent management operations).

Our results are compatible with the following interpretation: ID pigs in LS or HS conditions show a similar profile in the discriminant analysis, indicating that they perceive and respond similarly to the stress factors of transport, regardless of its severity. On the contrary, OD pigs show a very different profile in the discriminant analysis, indicating that they may cope well with a LS management, probably because they are already familiar with novel environments and human management procedures. But in HS conditions, the previous experience and cardiovascular fitness of OD pigs may not be enough to buffer a high stress situation.

Since no behavioural tests were carried out to correlate them to neurochemical responses, it is difficult to conclude what is “better” in the relationship between housing and transport-associated stress. Nevertheless, our results clearly show that the neurophysiological response of the pig is influenced by previous living conditions.

### Limitations of the study

The LS group was studied as the control group, although is not equivalent to the absence of transport stress. Nevertheless, this experimental design allows the comparison between two intensities of stress.

Another limitation is the exclusive use of female pigs, whereas several authors have reported gender-dependent effects in environmental enrichment studies in rodents [31,79,80]. Thus, further research is needed to test whether there are sex differences in the animal’s response in the porcine specie.

A third limitation might be in the interpretation of serum catecholamines. As blood samples were collected at exsanguination after the response to the transport conditions, the relationship with the housing conditions might be limited.

Finally, the present study was designed to analyze the neurophysiological differences caused by living and transport conditions in the pigs. Behavioural tests carried out in parallel will be necessary to reach conclusions that may affect policy guidelines for the care and handling of pigs.

### Conclusion

Our work showed that housing conditions and transport-associated stress modify the neurophysiology of pigs, shedding light about the molecular mechanisms underlying these changes. Altogether, our results indicate that the animals raised partially outdoors respond differently to transport-associated stress than animals raised indoors, suggesting that they cope differently with unknown environments.

Using an original approach, our results confirmed that external sensory inputs from the EE affect the pig brain. It is possible that, as suggested by van de Weerd [76], brain parameters could one day be useful tools to evaluate the effects of environmental enrichment. However, more work should be done to interpret the meaning of these effects.

## Supporting information

S1 Table Raw data. Data from serum biochemistry and catecholamines, PFC and HC monoamine NT and molecular markers of stress, and hippocampal BDNF of each animal in the study by housing, transport and pen.
(XLSX)

S2 Table Discriminant analysis. Wilks Lambda, Eigenvalues, cumulative proportions and standardized canonical discriminant function coefficients of the discriminant analysis. Only Discriminant functions 1 (DF1) and 2 (DF2) had eigenvalues > 1 and were included in the analysis.
(XLSX)
